# Five‐year outcomes of pembrolizumab versus chemotherapy in Chinese patients with non‐small‐cell lung cancer and programmed cell death ligand 1 tumor proportion score ≥1%: KEYNOTE‐042 China study

**DOI:** 10.1002/ijc.70265

**Published:** 2025-12-31

**Authors:** Yi‐Long Wu, Li Zhang, Yun Fan, JianYing Zhou, Li Zhang, Qing Zhou, Wei Li, ChengPing Hu, GongYan Chen, Xin Zhang, CaiCun Zhou, Carmen González Arenas, Zhenghong Chen, Wen Cheng Yu, Tony S. K. Mok

**Affiliations:** ^1^ Guangdong Lung Cancer Institute, Guangdong Provincial People's Hospital (Guangdong Academy of Medical Sciences) Southern Medical University Guangzhou China; ^2^ Respiratory Medicine Department Peking Union Medical College Hospital Beijing China; ^3^ Department of Thoracic Medical Oncology The Cancer Hospital of the University of Chinese Academy of Sciences (Zhejiang Cancer Hospital) Hangzhou China; ^4^ Department of Respiratory Medicine The First Affiliated Hospital of Zhejiang University Hangzhou China; ^5^ Department of Medical Oncology Sun Yat‐Sen University Cancer Center Guangzhou China; ^6^ Oncology Department The First Hospital of Jilin University Changchun China; ^7^ Department of Respiratory Medicine Xiangya Hospital–Central South University Changsha China; ^8^ Department of Respiratory Medicine The Third Affiliated Hospital of Harbin Medical University Harbin China; ^9^ Respiratory Diseases Department Zhongshan Hospital, Fudan University Shanghai China; ^10^ School of Medicine Tongji University and Shanghai Pulmonary Hospital Shanghai China; ^11^ Oncology European Clinical Development, Merck Research Laboratories, GCD MSD Spain Madrid Spain; ^12^ Medical Oncology MSD China Beijing China; ^13^ State Key Laboratory of Translational Oncology Chinese University of Hong Kong Hong Kong China

**Keywords:** chemotherapy, non‐small‐cell lung cancer, pembrolizumab, programmed cell death ligand 1

## Abstract

In the phase 3 KEYNOTE‐042 China study of participants enrolled in China in the global KEYNOTE‐042 (NCT02220894) and China extension (NCT03850444) studies, pembrolizumab improved overall survival (OS) versus chemotherapy in locally advanced or metastatic non‐small‐cell lung cancer (NSCLC) with programmed cell death ligand 1 (PD‐L1) tumor proportion score (TPS) ≥50% (hazard ratio [HR], 0.63; 95% CI, 0.43–0.94), ≥20% (0.66; 0.47–0.92), and ≥1% (0.67; 0.50–0.89). We present outcomes from this study after 5 years of follow‐up. Chinese participants with previously untreated locally advanced or metastatic NSCLC with PD‐L1 TPS ≥1% without *EGFR* or *ALK* alterations were eligible. Participants were randomized 1:1 to pembrolizumab 200 mg every 3 weeks for up to 35 cycles or carboplatin plus paclitaxel or pemetrexed with optional pemetrexed maintenance (nonsquamous only). Primary endpoints were OS in the PD‐L1 TPS ≥50%, ≥20%, and ≥1% subgroups. Median follow‐up was 63.7 (range, 56.3–72.6) months among 262 participants (pembrolizumab, *n* = 128; chemotherapy, *n* = 134) included in this study. Pembrolizumab prolonged OS versus chemotherapy in participants with PD‐L1 TPS ≥50% (HR, 0.65; 95% CI, 0.45–0.93), ≥20% (0.67; 0.49–0.91), and ≥1% (0.66; 0.51–0.87). Grade 3 to 5 treatment‐related AEs occurred in 19.5% and 68.8% of participants in the pembrolizumab and chemotherapy groups, respectively. In conclusion, after 5 years of follow‐up, pembrolizumab continued to demonstrate improved OS versus chemotherapy with manageable safety in Chinese participants with previously untreated locally advanced or metastatic NSCLC that expressed PD‐L1. These data further support pembrolizumab monotherapy as a standard of care for these patients.

## Graphical abstract

What's new?

Global research has found that for people newly diagnosed with advanced non‐small cell lung cancer (NSCLC), the anti‐PD‐1 monoclonal antibody pembrolizumab on its own often leads to better results than traditional chemotherapy. The present report details long‐term findings of pembrolizumab therapy specifically among Chinese patients. Data show that more than half of patients with previously untreated NSCLC who finished the full course of pembrolizumab were still alive 3 years later—roughly 5 years after they first joined the study. Though immune‐mediated adverse events were more common, patients taking pembrolizumab also experienced fewer treatment‐related side effects than those receiving chemotherapy.

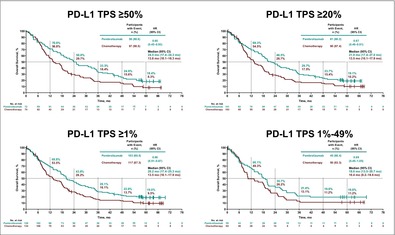

**Abbreviations**
AE: adverse event
BICR: blinded independent central review
CI: confidence interval
CR: complete response
DOR: duration of response
ECOG: Eastern Cooperative Oncology Group
HR: hazard ratio
ITT: intent to treat
NSCLC: non~‐small‐cell lung cancer
ORR: objective response rate
OS: overall survival
PD: progressive disease
PD‐1: programmed cell death protein 1
PD‐L1: programmed cell death ligand 1
PFS: progression‐free survival
PR: partial response
Q3W: every 3 weeks
RECIST: Response Evaluation Criteria in Solid Tumors
SD: stable disease
TPS: tumor proportion score

## 1 INTRODUCTION

Lung cancer has the highest incidence and mortality among all cancers in China, with most patients presenting with advanced or metastatic disease at diagnosis.^1^, ^2^ Immunotherapy, including anti–programmed cell death protein 1 (PD‐1) and anti–programmed cell death ligand 1 (PD‐L1) antibodies, as monotherapy or in combination with other therapies, has become a standard of care for patients with metastatic non‐small‐cell lung cancer (NSCLC) without targetable genetic alterations worldwide, including in China.^3^, ^4^, ^5^, ^6^ One widely available therapy is the anti‐PD‐1 monoclonal antibody pembrolizumab, which is approved as monotherapy and in combination with chemotherapy.

As monotherapy, pembrolizumab significantly improved overall survival (OS) versus chemotherapy with manageable safety in participants with previously untreated locally advanced or metastatic NSCLC with PD‐L1 tumor proportion score (TPS) ≥50% and without *EGFR* or *ALK* alterations in the global, phase 3 KEYNOTE‐024 study,^7^ and in participants with PD‐L1 TPS ≥1% in the global, phase 3 KEYNOTE‐042 study.^8^ Similar outcomes were observed in the KEYNOTE‐042 China study, which included participants enrolled in China from the KEYNOTE‐042 global and China extension studies.^9^ In the KEYNOTE‐042 China study, pembrolizumab monotherapy improved OS versus chemotherapy in participants with previously untreated locally advanced or metastatic NSCLC with PD‐L1 TPS ≥50% (HR, 0.63; 95% CI, 0.43–0.94), TPS ≥20% (0.66; 0.47–0.92), and TPS ≥1% (0.67; 0.50–0.89), with manageable toxicity.^9^

Pembrolizumab monotherapy has shown long‐term benefit in treatment outcomes compared with chemotherapy in participants with previously untreated advanced or metastatic NSCLC in global study populations.^10^, ^11^ However, similar analyses are not available specifically in Chinese patients. We present efficacy and safety outcomes from the KEYNOTE‐042 China study after approximately 5 years of follow‐up, including data for participants who completed 35 cycles (approximately 2 years) of pembrolizumab.

## 2 MATERIALS AND METHODS

### 2.1 Study design and participants

The KEYNOTE‐042 global (NCT02220894) and China extension (NCT0385044) study designs were similar and have been previously described.^8^, ^9^ The China extension study included only participants enrolled in mainland China after enrollment in the global study was completed. Eligible participants were at least 18 years of age with previously untreated advanced or metastatic NSCLC of any histology without targetable *EGFR* or *ALK* alterations, PD‐L1 TPS ≥1%, measurable disease per Response Evaluation Criteria in Solid Tumors (RECIST) version 1.1, life expectancy of at least 3 months, and an Eastern Cooperative Oncology Group (ECOG) performance status of 0 or 1.

Participants were randomized 1:1 to receive pembrolizumab 200 mg every 3 weeks (Q3W) for up to 35 cycles or carboplatin area under the curve of 5 or 6 mg/mL/min plus investigator's choice of paclitaxel 200 mg/m^2^ or pemetrexed 500 mg/m^2^ (nonsquamous histology only) Q3W for up to 4 to 6 cycles. Pemetrexed maintenance therapy was encouraged for participants with nonsquamous tumors. Stratification factors for randomization included ECOG performance status (0 vs. 1), histology (squamous vs. nonsquamous), and PD‐L1 TPS (≥50% vs. 1%–49%). Treatment continued for the maximum treatment cycles permitted per protocol or until progressive disease (PD) per RECIST version 1.1 as determined by blinded independent central review (BICR), intolerable toxicity, or participant withdrawal. Participants from the chemotherapy group were not allowed to cross over to receive pembrolizumab on study.

Participants randomized to the pembrolizumab group were eligible to receive a second course of pembrolizumab (up to 17 cycles) upon PD after either completing 35 cycles of pembrolizumab with stable disease (SD) or better or having achieved complete response (CR) after at least 6 months of treatment and a minimum of 2 cycles of pembrolizumab after CR assessment.

### 2.2 Outcomes

The primary endpoints of the study were OS, defined as the time from randomization to death from any cause, in participants with PD‐L1 TPS ≥50%, ≥20%, and ≥1%. The secondary endpoints included progression‐free survival (PFS), defined as the time from randomization to disease progression per RECIST version 1.1 by BICR or death from any cause, whichever occurred first; objective response rate (ORR), defined as the proportion of participants who have CR or partial response (PR) per RECIST version 1.1 by BICR in participants with PD‐L1 TPS ≥50%, ≥20%, and ≥1%; and safety. Exploratory endpoints included OS and PFS in participants with PD‐L1 TPS 1%–49%; duration of response (DOR) per RECIST version 1.1 by BICR in participants with PD‐L1 TPS ≥50%, ≥20%, and ≥1%; and progression‐free survival 2 (PFS2), defined as the time from randomization to subsequent disease progression per RECIST version 1.1 by investigator review after initiation of new anticancer therapy or death from any cause.

### 2.3 Assessments

Programmed cell death ligand 1 expression was assessed in a central laboratory using PD‐L1 IHC 22C3 pharmDx (Agilent Technologies, Carpinteria, CA, USA). Tumor imaging was performed at baseline, every 9 weeks from randomization until week 45, and every 12 weeks thereafter. Adverse events (AEs) were monitored from randomization to 30 days after the end of treatment (90 days for serious AEs) or until initiation of new anticancer therapy and were graded according to National Cancer Institute Common Terminology Criteria for Adverse Events version 4.0. Immune‐mediated AEs and infusion reactions were based on a list of preferred terms intended to capture known risks of pembrolizumab and were considered regardless of attribution to study treatment by the investigator.

### 2.4 Statistical analysis

Efficacy was assessed in the intent‐to‐treat population in participants with PD‐L1 TPS ≥50%, ≥20%, and ≥1%. Safety was assessed in the as‐treated population, defined as all randomized participants who received at least 1 dose of study treatment. The Kaplan–Meier method was used to estimate OS, PFS, and PFS2. A stratified Cox proportional hazards model with the Efron method of tie handling was used to determine the magnitude of the treatment differences (HRs and 95% CIs). The randomization stratification factors were applied to the stratified Cox model. Between‐group differences in ORR were analyzed using the stratified Miettinen and Nurminen method. There was no alpha assigned to this analysis.

## 3 RESULTS

### 3.1 Participants and treatment

The intent‐to‐treat population included 262 participants from China who were randomly assigned to pembrolizumab (*n* = 128) or chemotherapy (*n* = 134). Participant demographics and baseline clinical characteristics were generally similar between the treatment groups (Table 1). Most participants were men, had an ECOG performance status of 1, were current or former smokers, and had PD‐L1 TPS ≥50%.

**TABLE 1:** Participant demographics and disease characteristics at baseline.

Characteristic	ITT population (PD‐L1 TPS ≥1%)		Completed 35 cycles of pembrolizumab (PD‐L1 TPS ≥1%) *n* = 22^a^
	Pembrolizumab *n* = 128	Chemotherapy *n* = 134	
Age, median (range), year	62.0 (22–78)	62.0 (32–82)	61.5 (52–72)
Sex			
Men	105 (82.0)	119 (88.8)	19 (86.4)
Women	23 (18.0)	15 (11.2)	3 (13.6)
ECOG performance status			
0	31 (24.2)	29 (21.6)	7 (31.8)
1	97 (75.8)	105 (78.4)	15 (68.2)
Smoking status			
Current/former	97 (75.8)	106 (79.1)	19 (86.4)
Never	31 (24.2)	28 (20.9)	3 (13.6)
Tumor histology			
Squamous	71 (55.5)	76 (56.7)	12 (54.5)
Nonsquamous	57 (44.5)	58 (43.3)	10 (45.5)
Disease status			
Metastatic	106 (82.8)	102 (76.1)	17 (77.3)
Locally advanced	22 (17.2)	32 (23.9)	5 (22.7)
Brain metastasis	2 (1.6)	2 (1.5)	1 (4.5)
PD‐L1 TPS			
≥50%	72 (56.3)	74 (55.2)	18 (81.8)
20%–49%	29 (22.7)	29 (21.6)	2 (9.1)
1%–19%	27 (21.1)	31 (23.1)	2 (9.1)
Prior treatment			
Radiotherapy	6 (4.7)	4 (3.0)	2 (9.1)
(Neo)adjuvant therapy	9 (7.0)	2 (1.5)	0

*Note*: Data are *n* (%) unless otherwise noted. Some data were previously published in Wu et al.^9^

Abbreviations: ECOG, Eastern Cooperative Oncology Group; ITT, intent to treat; PD‐L1, programmed cell death ligand 1; TPS, tumor proportion score.

^a^ One participant completed 35 cycles of pembrolizumab despite experiencing progressive disease per blinded independent central review.

Median time from randomization to database cutoff (September 12, 2022) was 63.7 (range, 56.3–72.6) months. At the time of database cutoff, 21 participants (16.4%) in the pembrolizumab group and 34 (27.2%) in the chemotherapy group had completed treatment; no participants remained on the originally assigned treatment. Eighty participants (62.5%) in the pembrolizumab group received subsequent anticancer therapy as of the database cutoff, including 10 (7.8%) who received subsequent immunotherapy; an additional five participants began second‐course pembrolizumab (Table S1). In the chemotherapy group, 79 participants (59.0%) received subsequent anticancer therapy, including 35 (26.1%) who received subsequent immunotherapy.

### 3.2 Efficacy outcomes in the intent‐to‐treat population

**FIGURE 1:**
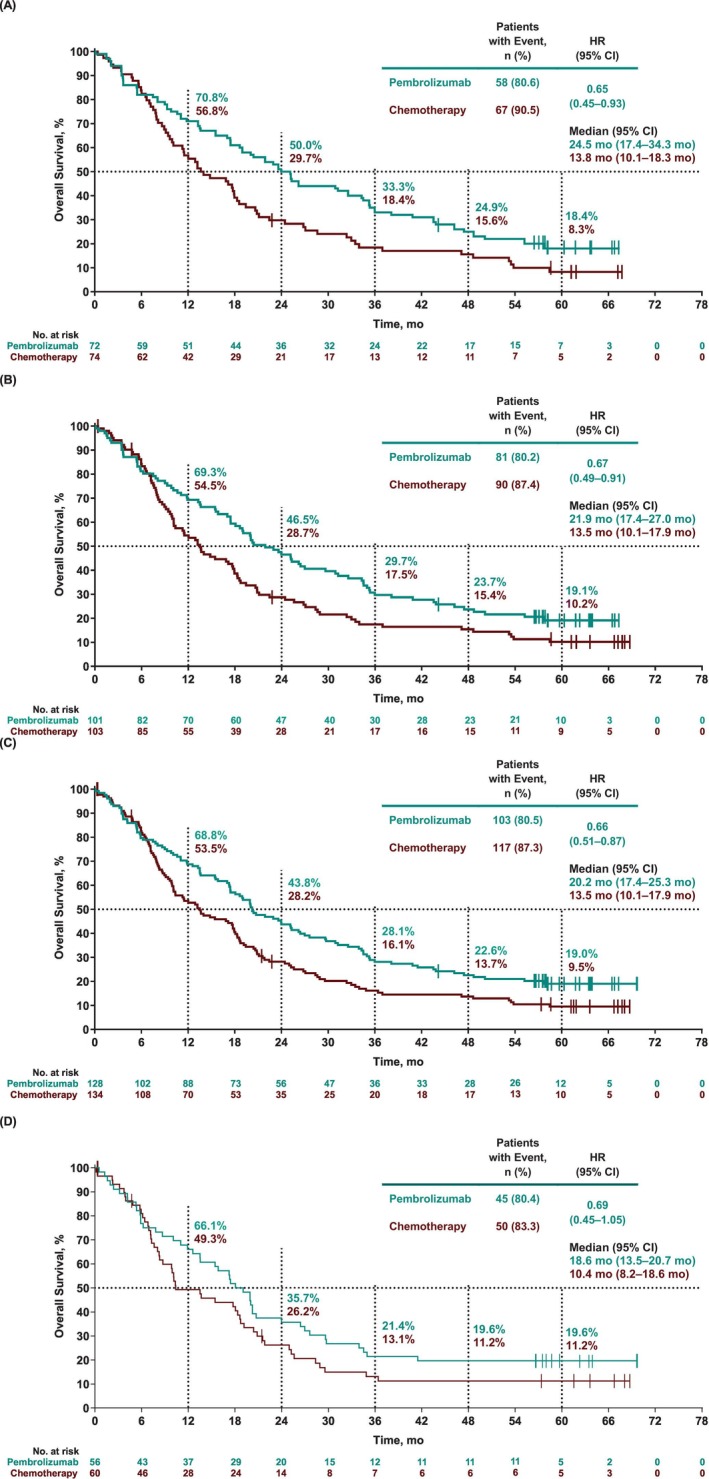
Kaplan–Meier estimates of overall survival among participants with PD‐L1 TPS (A) ≥50%, (B) ≥20%, (C) ≥1%, and (D) 1%–49%. HR, hazard ratio; NR, not reached; PD‐L1, programmed cell death ligand 1; TPS, tumor proportion score.

Median OS for pembrolizumab versus chemotherapy was 24.5 months versus 13.8 months in participants with PD‐L1 TPS ≥50%, 21.9 months versus 13.5 months in participants with TPS ≥20%, and 20.2 months versus 13.5 months in participants with TPS ≥1% (Figure 1A–C). The OS HR for pembrolizumab versus chemotherapy was 0.65 (95% CI, 0.45–0.93) in the PD‐L1 TPS ≥50% subgroup, 0.67 (0.49–0.91) in the PD‐L1 TPS ≥20% subgroup, and 0.66 (0.51–0.87) in the PD‐L1 TPS ≥1% subgroup. The estimated 5‐year OS rates were 18.4% (95% CI, 10.2%–28.5%) for pembrolizumab and 8.3% (3.3%–16.2%) for chemotherapy in the PD‐L1 TPS ≥50% subgroup; 19.1% (12.0%–27.5%) and 10.2% (5.2%–17.1%), respectively, in the PD‐L1 TPS ≥20% subgroup; and 19.0% (12.6%–26.4%) and 9.5% (5.2%–15.5%), respectively, in the PD‐L1 TPS ≥1% subgroup. In a prespecified exploratory analysis of participants with PD‐L1 TPS 1%–49%, median OS was 18.6 months in the pembrolizumab group and 10.4 months in the chemotherapy group (Figure 1D); the HR was 0.69 (95% CI, 0.45–1.05). The estimated 5‐year OS rates in this population were 19.6% (95% CI, 10.5%–30.9%) and 11.2% (4.6%–21.1%) in the pembrolizumab and chemotherapy groups, respectively (Figure 1D). Across most participant subgroups, HRs for OS favored pembrolizumab over chemotherapy (Figure S1).

Median PFS for pembrolizumab versus chemotherapy was 8.3 versus 6.6 months in participants with PD‐L1 TPS ≥50%, 6.3 versus 7.5 months in participants with PD‐L1 TPS ≥20%, and 6.4 versus 6.8 months in participants with PD‐L1 TPS ≥1% (Table 2 and Figure S2). The HR for PFS for pembrolizumab versus chemotherapy was 0.94 (95% CI, 0.67–1.34) in participants with PD‐L1 TPS ≥50%, 1.03 (0.76–1.38) in participants with PD‐L1 TPS ≥20%, and 1.04 (0.80–1.35) in participants with PD‐L1 TPS ≥1%. In the PD‐L1 TPS 1%–49% subgroup, median PFS was 6.0 (95% CI, 4.0–6.5) months versus 7.2 (5.9–10.2) months in the pembrolizumab versus chemotherapy groups; the HR was 1.18 (95% CI, 0.79–1.77).

**TABLE 2:** Summary of PFS and PFS2 by PD‐L1 TPS.

Variable	PD‐L1 TPS ≥50%		PD‐L1 TPS ≥20%		PD‐L1 TPS ≥1%	
	Pembrolizumab (*n* = 72)	Chemotherapy (*n* = 74)	Pembrolizumab (*n* = 101)	Chemotherapy (*n* = 103)	Pembrolizumab (*n* = 128)	Chemotherapy (*n* = 134)
PFS^a^						
Median (95% CI), month	8.3 (4.2–13.1)	6.6 (4.4–11.4)	6.3 (4.2–9.0)	7.5 (6.0–9.9)	6.4 (4.2–8.3)	6.8 (6.0–9.7)
5‐year rate (95% CI), %	7.7 (2.9–15.6)	2.9 (0.5–9.0)	7.6 (3.4–14.1)	2.1 (0.4–6.7)	6.3 (2.8–11.7)	1.6 (0.3–5.3)
HR (95% CI)	0.94 (0.67–1.34)		1.03 (0.76–1.38)		1.04 (0.80–1.35)	
PFS2^b^						
Median (95% CI), month	16.8 (11.5–21.9)	9.7 (7.9–13.2)	15.1 (11.5–19.2)	9.7 (7.9–11.3)	14.3 (11.1–17.1)	8.9 (7.8–10.7)
5‐year rate (95% CI), %	11.1 (5.2–19.6)	3.1 (0.6–9.6)	9.9 (5.1–16.6)	2.2 (0.4–7.0)	9.6 (5.2–15.6)	2.2 (0.5–6.4)
HR (95% CI)	0.58 (0.40–0.83)		0.58 (0.43–0.79)		0.59 (0.45–0.78)	

Abbreviations: PD‐L1, programmed cell death ligand 1; PFS, progression‐free survival; RECIST, Response Evaluation Criteria in Solid Tumors; TPS, tumor proportion score.

^a^ All responses were confirmed and are based on RECIST version 1.1 by blinded independent central review.

^b^ Defined as the time from randomization to second or subsequent radiographic progressive disease per RECIST version 1.1 by investigator review on next‐line treatment or death from any cause, whichever came first.

The ORR for pembrolizumab versus chemotherapy was 41.7% (95% CI, 30.2%–53.9%) versus 24.3% (15.1%–35.7%) in participants with PD‐L1 TPS ≥50%, 34.7% (25.5%–44.8%) versus 24.3% (16.4%–33.7%) in participants with PD‐L1 TPS ≥20%, and 32.0% (24.1%–40.9%) versus 24.6% (17.6%–32.8%) in participants with PD‐L1 TPS ≥1% (Table 3). Median DOR for pembrolizumab versus chemotherapy was 16.5 (range, 1.4+ to 64.1+) months versus 11.7 (1.6+ to 63.4+) months in participants with PD‐L1 TPS ≥50%, 16.5 (1.4+ to 64.1+) months versus 10.9 (1.6+ to 63.4+) months in participants with PD‐L1 TPS ≥20%, and 16.0 (1.4+ to 64.1+) months versus 10.9 (1.1+ to 63.4+) months in participants with PD‐L1 TPS ≥1% (“+” indicates that the response was ongoing at the last disease assessment before data cutoff).

**TABLE 3:** Tumor response per RECIST version 1.1 by BICR.

	ITT population						Participants who completed 35 cycles of pembro (PD‐L1 TPS ≥1%)
	PD‐L1 TPS ≥50%		PD‐L1 TPS ≥20%		PD‐L1 TPS ≥1%		
	Pembro, *n* = 72	Chemo, *n* = 74	Pembro, *n* = 101	Chemo, *n* = 103	Pembro, *n* = 128	Chemo, *n* = 134	*n* = 22
ORR (95% CI),^a^ %	41.7 (30.2–53.9)	24.3 (15.1–35.7)	34.7 (25.5–44.8)	24.3 (16.4–33.7)	32.0 (24.1–40.9)	24.6 (17.6–32.8)	81.8 (59.7–94.8)
Best overall response, *n* (%)							
CR	0	1 (1.4)	0	1 (1.0)	0	1 (0.7)	0
PR	30 (41.7)	17 (23.0)	35 (34.7)	24 (23.3)	41 (32.0)	32 (23.9)	18 (81.8)
SD	21 (29.2)	31 (41.9)	35 (34.7)	46 (44.7)	47 (36.7)	56 (41.8)	4 (18.2)
PD	16 (22.2)	12 (16.2)	23 (22.8)	15 (14.6)	31 (24.2)	20 (14.9)	0
NE^b^	1 (1.4)	0	3 (3.0)	1 (1.0)	4 (3.1)	2 (1.5)	0
NA^c^	4 (5.6)	13 (17.6)	5 (5.0)	16 (15.5)	5 (3.9)	23 (17.2)	0
DOR,^d^ median (range), month	16.5 (1.4+ to 64.1+)	11.7 (1.6+ to 63.4+)	16.5 (1.4+ to 64.1+)	10.9 (1.6+ to 63.4+)	16.0 (1.4+ to 64.1+)	10.9 (1.1+ to 63.4+)	33.6 (6.1 to 64.1+)
DOR ≥3 years, no. at risk (%)	5 (23.1)	2 (34.0)	6 (25.3)	2 (25.6)	7 (25.9)	2 (16.6)	—

Abbreviations: BICR, blinded independent central review; Chemo, chemotherapy; CR, complete response; DOR, duration of response; ITT, intent to treat; NA, no assessment; NE, not evaluable; ORR, objective response rate; PD, progressive disease; PD‐L1, programmed cell death protein 1; Pembro, pembrolizumab; PR, partial response; RECIST, Response Evaluation Criteria in Solid Tumors; SD, stable disease; TPS, tumor proportion score; +, no PD by the time of last assessment.

^a^ Per RECIST version 1.1 by investigator central review.

^b^ Postbaseline assessment(s) available but not evaluable or CR/PR/SD <6 weeks from randomization.

^c^ No postbaseline assessment available for response evaluation.

^d^ Per Kaplan–Meier estimate.

Median PFS2 for pembrolizumab versus chemotherapy was 16.8 versus 9.7 months in participants with PD‐L1 TPS ≥50%, 15.1 versus 9.7 months in participants with PD‐L1 TPS ≥20%, and 14.3 versus 8.9 months in participants with PD‐L1 TPS ≥1% (Table 2 and Figure S3). The HR was 0.58 (95% CI, 0.40–0.83) in participants with PD‐L1 TPS ≥50%, 0.58 (0.43–0.79) in participants with PD‐L1 TPS ≥20%, and 0.59 (0.45–0.78) in participants with PD‐L1 TPS ≥1%.

### 3.3 Safety

In the as‐treated population, median (range) treatment duration for participants in the pembrolizumab group (first course only) was 6.4 months (0.03–25.4) and in the chemotherapy group was 3.1 months (0.03–56.7). Among the as‐treated population, treatment‐related AEs occurred in 106 of 128 participants (82.8%) in the pembrolizumab group and 115 of 125 participants (92.0%) in the chemotherapy group; 25 (19.5%) and 86 (68.8%) participants, respectively, had grade 3 to 5 events (Table 4). Treatment‐related AEs led to death in 8 participants (6.3%) in the pembrolizumab group and 4 participants (3.2%) in the chemotherapy group. All of these grade 5 treatment‐related AEs were previously reported,^9^ with the exception of 1 additional participant with malignant neoplasm progression in the pembrolizumab group (Table 4). In total, 20 participants (15.6%) in the pembrolizumab group and 18 (14.4%) in the chemotherapy group discontinued treatment due to treatment‐related AEs. Exposure‐adjusted treatment‐related AE rates (Table S2) generally decreased over time in both treatment groups.

**TABLE 4:** Adverse events in the as‐treated population.^a^

Adverse event, *n* (%)	Pembrolizumab (*n* = 128)		Chemotherapy (*n* = 125)	
Any treatment‐related adverse event	106 (82.8)		115 (92.0)	
Grade 3–5	25 (19.5)		86 (68.8)	
Led to treatment discontinuation	20 (15.6)		18 (14.4)	
Led to death	8 (6.3)^b^		4 (3.2)^c^	
Occurring in ≥10% of participants in either treatment group	**Any Grade**	**Grade 3–5**	**Any Grade**	**Grade 3–5**
Increased alanine aminotransferase	22 (17.2)	2 (1.6)	27 (21.6)	1 (0.8)
Increased aspartate aminotransferase	22 (17.2)	1 (0.8)	23 (18.4)	1 (0.8)
Rash	17 (13.3)	1 (0.8)	5 (4.0)	0
Hypothyroidism	15 (11.7)	0	0	0
Pyrexia	15 (11.7)	1 (0.8)	10 (8.0)	0
Pruritus	14 (10.9)	0	2 (1.6)	0
Anemia	11 (8.6)	1 (0.8)	65 (52.0)	21 (16.8)
Fatigue	8 (6.3)	0	20 (16.0)	1 (0.8)
Decreased appetite	7 (5.5)	0	36 (28.8)	3 (2.4)
Decreased platelet count	4 (3.1)	0	28 (22.4)	7 (5.6)
Decreased neutrophil count	3 (2.3)	0	70 (56.0)	45 (36.0)
Leukopenia	3 (2.3)	0	16 (12.8)	8 (6.4)
Constipation	2 (1.6)	0	17 (13.6)	0
Nausea	2 (1.6)	0	23 (18.4)	1 (0.8)
Alopecia	1 (0.8)	0	32 (25.6)	1 (0.8)
Decreased white blood cell count	1 (0.8)	0	64 (51.2)	22 (17.6)
Vomiting	1 (0.8)	0	16 (12.8)	1 (0.8)
Neutropenia	0	0	14 (11.2)	6 (4.8)
Immune‐mediated adverse events and infusion reactions	34 (26.6)	9 (7.0)	7 (5.6)	4 (3.2)
Led to death	1 (0.8)	1 (0.8)^d^	0	0
Hypothyroidism	15 (11.7)	0	0	0
Pneumonitis	10 (7.8)	3 (2.3)	0	0
Hyperthyroidism	7 (5.5)	1 (0.8)	0	0
Infusion reactions	4 (3.1)	1 (0.8)	7 (5.6)	4 (3.2)
Hepatitis	2 (1.6)	2 (1.6)	0	0
Severe skin reactions	2 (1.6)	1 (0.8)	0	0
Thyroiditis	2 (1.6)	0	0	0
Colitis	1 (0.8)	0	0	0
Hypoparathyroidism	1 (0.8)	0	0	0
Pancreatitis	1 (0.8)	1 (0.8)	0	0

*Note*: Some data were previously published in Wu et al.^9^

^a^ The as‐treated population comprised all randomized participants who received at least 1 dose of study treatment, according to the treatment received.

^b^ Malignant neoplasm progression, *n* = 2; acute left ventricular failure, myocardial infarction, multiple organ dysfunction syndrome, pneumonia, interstitial lung disease, and pulmonary embolism, *n* = 1 each.

^c^ Septic shock, *n* = 2; ketoacidosis and pulmonary embolism, *n* = 1 each.

^d^ One grade 5 event of pneumonitis.

Immune‐mediated AEs and infusion reactions were irrespective of treatment attribution by the investigator and occurred in 34 participants (26.6%) in the pembrolizumab group and seven participants (5.6%) in the chemotherapy group. Eight participants (6.3%) experienced grade 3 events in the pembrolizumab group; there was one grade 5 event of pneumonitis that was previously reported.^9^ Four participants (3.2%) in the chemotherapy group experienced grade 3 or 4 events (Table 4). Exposure‐adjusted immune‐mediated AE and infusion reaction rates decreased over time in both treatment groups (Table S3).

### 3.4 Outcomes in participants who completed 35 cycles of pembrolizumab

**FIGURE 2:**
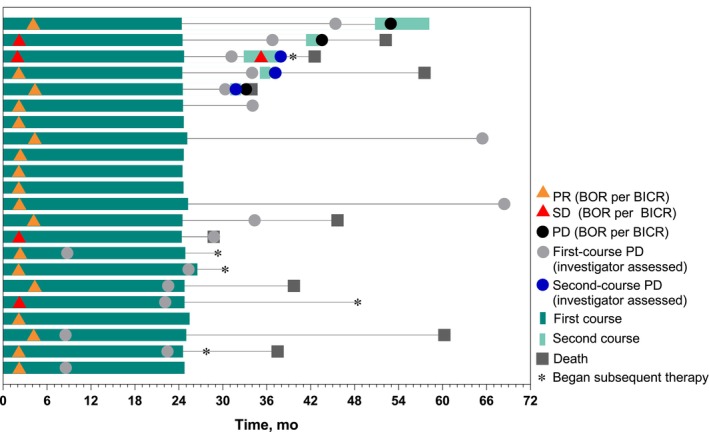
Treatment duration and time to response in participants who completed 35 cycles of pembrolizumab. BICR, blinded independent central review; BOR, best overall response; PD, progressive disease; PR, partial response; SD, stable disease.

Median time from completion of 35 cycles to database cutoff for the 22 participants who completed 35 cycles (approximately 2 years) of pembrolizumab was 39.5 (range, 32.7–47.1) months. ORR among participants who completed 35 cycles of pembrolizumab was 81.8%, and median DOR was 33.6 (range, 6.1–64.1+) months (Table 3). At database cutoff, 5 participants were alive without PD and subsequent therapy (Figure 2). Median OS from the time of completion of 35 cycles was not reached. The estimated 3‐year OS rate after completion of 35 cycles (i.e., approximately 5 years after randomization) was 56.6%.

Among participants who completed 35 cycles of pembrolizumab, treatment‐related AEs occurred in 17 participants (77.3%); there was one grade 3 event (ventricular arrhythmia) and no grade 4 events. Immune‐mediated AEs and infusion reactions occurred in 14 participants (36.4%); all were grade 1 or 2.

## 4 DISCUSSION

The 5‐year follow‐up from the KEYNOTE‐042 China study shows that pembrolizumab monotherapy improved survival outcomes compared with platinum‐based chemotherapy with manageable safety in Chinese participants with previously untreated locally advanced or metastatic NSCLC without targetable *EGFR* or *ALK* alterations and with PD‐L1 TPS ≥1%. OS and PFS2 were prolonged with pembrolizumab versus chemotherapy, and ORR and DOR were also improved with pembrolizumab versus chemotherapy among participants with PD‐L1 TPS ≥50%, ≥20%, and ≥1%. More than 50% of participants who completed 35 cycles of pembrolizumab were alive 3 years after completing treatment (approximately 5 years after randomization).

In this 5‐year follow‐up, there was a clear and consistent separation of the OS Kaplan–Meier curves favoring pembrolizumab over chemotherapy, which was maintained over time across all analyzed PD‐L1 TPS groups. This includes the exploratory PD‐L1 TPS 1%–49% subgroup, where the HR favored pembrolizumab versus chemotherapy. Additionally, the estimated 5‐year OS rates were nearly 2‐fold higher with pembrolizumab versus chemotherapy in all PD‐L1 TPS groups and, as expected, the largest difference was observed in participants with PD‐L1 TPS ≥50%. One limitation to be noted is that 10% or less of randomized participants were represented in the data at 5 years of follow‐up. However, this is not unusual for long‐term follow‐up analyses, and is similar to the 5‐year follow‐up of the KEYNOTE‐042 global study.^11^ While the smaller analysis population may impact the robustness of the findings from this study, the OS findings presented clearly demonstrate the benefits of pembrolizumab compared with chemotherapy after 5 years of follow‐up.

Despite the improvement in OS across PD‐L1 TPS groups, PFS was not improved with pembrolizumab versus chemotherapy. The dissociation between PFS and OS benefits, particularly at lower PD‐L1 TPS cut points, is well described for immunotherapy.^12^ Modest, or even absent, PFS or ORR benefit did not rule out long‐term OS benefit since imaging‐based end points may not fully capture the clinical benefit of immune checkpoint inhibition compared with the use of OS as an endpoint.^13^ HRs for PFS2 also favored pembrolizumab versus chemotherapy across all PD‐L1 TPS groups analyzed. These data further support the use of pembrolizumab as first‐line therapy compared with chemotherapy because no on‐study crossover was allowed from the chemotherapy group to pembrolizumab monotherapy.

Objective response rate and DOR were improved in the pembrolizumab group compared with the chemotherapy group in all PD‐L1 TPS groups examined. These data are consistent with previous findings from this study^9^ and continue to demonstrate that participants in the pembrolizumab group who experienced an objective response had durable tumor responses. Consistent with these findings, more than 80% of the 22 participants who completed 35 cycles of pembrolizumab experienced an objective response, with a median DOR of nearly 3 years. The estimated 3‐year OS rate after completion of 35 cycles of pembrolizumab (approximately 5 years after randomization) was more than 50% and demonstrates prolonged treatment benefit after 2 years of pembrolizumab monotherapy.

The toxicity profile was similar to previously reported safety data from this study.^9^ The incidence of treatment‐related AEs of any grade was lower in the pembrolizumab group than in the chemotherapy group, and the incidence of grade 3 to 5 events was substantially lower with pembrolizumab than with chemotherapy (19.5% vs. 68.8%). These data are similar to those observed in the KEYNOTE‐042 global study.^8^, ^11^ The incidence of immune‐mediated AEs and infusion reactions was higher in the pembrolizumab group versus the chemotherapy group, which is expected given the mechanism of action of pembrolizumab, and was similar to the incidence of these events reported in the global study population.^11^

In conclusion, the outcomes of this 5‐year follow‐up extend the findings observed in the previous analysis of the KEYNOTE‐042 China study and are similar to those observed in the 5‐year follow‐up from the KEYNOTE‐042 global study.^11^ The results presented here continue to demonstrate improved OS and durable responses with pembrolizumab versus chemotherapy and manageable safety in Chinese participants with previously untreated locally advanced or metastatic NSCLC with PD‐L1 TPS ≥50%, ≥20%, and ≥1% without targetable *EGFR* or *ALK* alterations. Consistent with the global KEYNOTE‐042 study, these results continue to support the use of pembrolizumab monotherapy as a standard of care in this population.

## AUTHOR CONTRIBUTIONS

**Yi‐Long Wu:** Conceptualization; data curation; writing – review and editing; writing – original draft. **Li Zhang:** Data curation; writing – review and editing; data curation; writing – review and editing. **Yun Fan:** Data curation; writing – review and editing. **JianYing Zhou:** Data curation; writing – review and editing. **Li Zhang:** Data curation; writing – review and editing; data curation; writing – review and editing. **Qing Zhou:** Conceptualization; writing – review and editing. **Wei Li:** Data curation; writing – review and editing. **ChengPing Hu:** Data curation; writing – review and editing. **GongYan Chen:** Data curation; writing – review and editing. **Xin Zhang:** Data curation; writing – review and editing. **CaiCun Zhou:** Data curation; writing – review and editing. **Carmen González Arenas:** Data curation; writing – original draft; writing – review and editing. **Zhenghong Chen:** Data curation; writing – review and editing. **Wen Cheng Yu:** Data curation; writing – review and editing. **Tony S. K. Mok:** Conceptualization; writing – original draft; writing – review and editing; data curation.

## CONFLICT OF INTEREST STATEMENT

Yi‐Long Wu: Grants and research support from AstraZeneca, Boehringer Ingelheim, BMS, Hengrui, and Roche; Advisor/Board member for AstraZeneca, Boehringer Ingelheim, Novartis, Takeda, and MSD; and Honorarium from AstraZeneca, Beigen, Boehringer Ingelheim, BMS, Eli Lilly, MSD, Novartis, Pfizer, Roche, and Sanofi (none were promotional activities). Li Zhang, Yun Fan, JianYing Zhou, Wei Li, ChengPing Hu, GongYan Chen, and Xin Zhang: Merck Sharp & Dohme LLC, a subsidiary of Merck & Co., Inc., Rahway, NJ, USA provided funding support for study conduct and medical writing. Li Zhang: Research grants from Hengrui, BMS, and Innovent Biologics. Qing Zhou: Honoraria from AstraZeneca, Lilly, Roche, Pfizer, Boehringer Ingelheim, MSD Oncology, Bristol‐Myers Squibb, Hengrui, and BeOne Medicines Ltd outside the submitted work. CaiCun Zhou: Honoraria from Boehringer Ingelheim, Eli Lilly, Hengrui, MSD, Sanofi, F. Hoffmann‐La Roche Ltd., and Qilu. Carmen González Arenas: Employee of MSD Spain and owns stock in Merck & Co., Inc., Rahway, NJ, USA. Zhenghong Chen and Wen Cheng Yu: Employees of MSD China. Tony S.K. Mok: Received grants or research support from AstraZeneca, Bristol Myers Squibb, G1 Therapeutics, MSD, Merck Serono, Novartis, Pfizer, Roche, SFJ Pharmaceuticals, and XCovery; speaker fees from AbbVie Inc., ACEA Pharma, Adagene, Alentis Therapeutics AG, Alpha Biopharma Co., Ltd., Amgen, Amoy Diagnostics Co., Ltd., AnHeart Therapeutics, AstraZeneca (before January 1, 2019), AVEO Pharmaceuticals, Inc., Bayer Healthcare Pharmaceuticals Ltd., BeiGene, BerGenBio ASA, Berry Oncology, Boehringer Ingelheim, Blueprint Medicines Corporation, BMS, Bowtie Life Insurance Company Limited, Bridge Biotherapeutics Inc., Covidien LP, C4 Therapeutics Inc., Cirina Ltd., CStone Pharmaceuticals, Curio Science, D3 Bio Ltd., Da Volterra, Daiichi Sankyo, Eisai, Elevation Oncology, F. Hoffmann‐La Roche Ltd., Genentech, GLG's Healthcare, Fishawack Facilitate Ltd., G1 Therapeutics Inc., geneDecode Co., Ltd, Gilead Sciences, Inc. Gritstone Oncology, Inc., Guardant Health, Hengrui Therapeutics Inc., HutchMed, Ignyta, Inc., Illumina, Inc., Incyte Corporation, Inivata, InxMed (Hong Kong) Limited, IQVIA, Janssen, Lakeshore Biotech Ltd, Lilly, Lunit USA, Inc., Loxo‐Oncology, Lucence Health Inc., Medscape LLC/WebMD, Medtronic, Merck Serono, MSD, Mirati Therapeutics Inc., MiRXES, MoreHealth, Ningbo NewBay Technology Development Co., Ltd, Novartis, Novocure GmbH, Omega Therapeutics Inc., OrigiMed, OSE Immunotherapeutics, Phanes Therapeutics, Inc., PeerVoice, Pfizer, PrIME Oncology, Prenetics, Puma Biotechnology Inc., Qiming Development (HK) Ltd., Regeneron Pharmaceuticals Inc., Roche Pharmaceuticals/Diagnostics/Foundation One, Sanofi‐Aventis, Schrödinger, Inc., SFJ Pharmaceutical Ltd., Simcere of America Inc., Summit Therapeutics Sub, Inc., Synergy Research, Takeda Pharmaceuticals HK Ltd., Tigermed, Vertex Pharmaceuticals, Virtus Medical Group, XENCOR, Inc., and Yuhan Corporation; is a stockholder in Alentis Therapeutics AG, AstraZeneca, Aurora Tele‐Oncology Ltd., Biolidics Ltd., HutchMed, Prenetics, D3 Bio, and Lunit Inc.; holds stock options in, Bowtie Life Insurance Co. Ltd., D3 Bio, Lakeshore Biotech Ltd, Loxo‐oncology, Lunit USA, Inc., Virtus Medical Group, Phanes Therapeutics, Inc., and Insighta; served as an advisory board member forAbbVie Inc., ACEA Pharma, Alentis Therapeutics AG, Amgen, AstraZeneca, BerGenBio ASA, Berry Oncology, Blueprint Medicines Corporation, Boehringer Ingelheim, Bowtie Life Insurance Co Ltd, Bristol Myers Squibb, C4 Therapeutics Inc, Covidien LP, CStone Pharmaceuticals, Curio Science, D3 Bio Ltd., Daiichi Sankyo Inc., Eisai, Fishawack Facilitate Ltd., G1 Therapeutics Inc., Gilead Sciences Inc., Gritstone Oncology, Inc., Guardant Health, geneDecode Co. Ltd. (uncompensated), Hengrui Therapeutics Inc., HutchMed, Ignyta Inc., Incyte Corporation, Imagene AI Ltd., Inivata, IQVIA, Janssen, Lakeshore Biotech, Lily, Loxo‐Oncology Inc., Lunit, Inc., Merck Serono, MSD, Mirati Therapeutics Inc., MiRXES Group, Novartis, OrigiMed, Pfizer, Prenetics, Puma Biotechnology Inc., Roche/Genentech, Regeneron Pharmaceuticals Inc., Sanofi‐Aventis R&D, SFJ Pharmaceutical, Simcere of America Inc., Simcere Zaiming, Inc., Summit Therapeutics, Inc., Takeda, Vertex Pharmaceuticals, Virtus Medical Group, and Yuhan Corporation; served on the board of directors or in a leadership role (renumerated) for AstraZeneca PLC (from January 2019 to present), HutchMed, Aurora, Insighta (from July 2023 to present), and Epoch (from April 2023 to present); and served on the board of directors or in a leadership role (non‐renumerated) for American Society of Clinical Oncology (ASCO) (2018–2022), Asian Thoracic Oncology Research Group (ATORG) (2016 to Present), Chinese Lung Cancer Research Foundation Limited (CLCRF) (2005–2012), Chinese Society of Clinical Oncology (CSCO) (2009–2020), Hong Kong Cancer Fund (HKCF) (2011 to Present), Hong Kong Cancer Therapy Society (HKCTS) (2004 to Present), International Association for the Study of Lung Cancer (IASLC) (2007–2019), St. Stephen's College & Prep. School (2017 to Present), and Hong Kong Academy of Sciences (ASHK) (2022 to Present).

## ETHICS STATEMENT

The study protocol was approved by institutional review boards/independent ethics committees at each site. All participants provided written informed consent.

## Supporting information


**Table S1.** Subsequent Therapies in the ITT Population (PD‐L1 TPS ≥ 1%).
**Table S2.** Exposure‐Adjusted AE Rates for Treatment‐Related AEs That Occurred in ≥ 10% of Participants in Either Treatment Group.
**Table S3.** Exposure‐Adjusted Treatment‐Related AE Rates for Immune‐Mediated AEs and Infusion Reactions.
**Figure S1.** OS Analysis in Key Subgroups in Participants With PD‐L1 TPS ≥ 1%.
**Figure S2.** Kaplan–Meier estimates of PFS in participants with (A) PD‐L1 TPS ≥ 50%, (B) PD‐L1 TPS ≥ 20%, and (C) PD‐L1 TPS ≥ 1%.
**Figure S3.** Kaplan–Meier estimates of PFS2 in participants with (A) PD‐L1 TPS ≥ 50%, (B) PD‐L1 TPS ≥ 20%, and (C) PD‐L1 TPS ≥ 1%.

## Data Availability

The data sharing policy, including procedures and restrictions, of Merck Sharp & Dohme LLC, a subsidiary of Merck & Co., Inc., Rahway, NJ, USA (MSD) is available at: https://externaldatasharing-msd.com/. Requests for access to the clinical study data can be submitted via email to the Data Access mailbox (dataaccess@msd.com).
