# Supplementary material for: Five‐year outcomes of pembrolizumab versus chemotherapy in Chinese patients with non‐small‐cell lung cancer and programmed cell death ligand 1 tumor proportion score ≥1%: KEYNOTE‐042 China study
Source: Int J Cancer. 2025 Dec 31;158(9):2429–39. doi: 10.1002/ijc.70265 (PMC12963706; doi:10.1002/ijc.70265)
Supplement: Supplementary file 1 — Table S1. Subsequent Therapies in the ITT Population (PD‐L1 TPS ≥ 1%). Table S2. Exposure‐Adjusted AE Rates for Treatment‐Related AEs That Occurred in ≥ 10% of Participants in Either Treatment Group. Table S3. Exposure‐Adjusted Treatment‐Related AE Rates for Immune‐Mediated AEs and Infusion Reactions. Figure S1. OS Analysis in Key Subgroups in Participants With PD‐L1 TPS ≥ 1%. Figure S2. Kaplan–Meier estimates of PFS in participants with (A) PD‐L1 TPS ≥ 50%, (B) PD‐L1 TPS ≥ 20%, and (C) PD‐L1 TPS ≥ 1%. Figure S3. Kaplan–Meier estimates of PFS2 in participants with (A) PD‐L1 TPS ≥ 50%, (B) PD‐L1 TPS ≥ 20%, and (C) PD‐L1 TPS ≥ 1%. [file IJC-158-2429-s001.pdf]

SUPPLEMENTAL MATERIAL

Five-Year Outcomes of Pembrolizumab versus Chemotherapy in Chinese Patients With Non–Small-Cell Lung Cancer and Programmed Cell Death Ligand 1 Tumor Proportion Score ≥1%: KEYNOTE-042 China Study

Yi-Long Wu, Li Zhang, Yun Fan, JianYing Zhou, Li Zhang, Qing Zhou, Wei Li, ChengPing Hu, GongYan Chen, Xin Zhang, CaiCun Zhou, Carmen González Arenas, Zhenghong Chen, Wen Cheng Yu, Tony S.K. Mok

Table of Contents

Table S1. Subsequent Therapies in the ITT Population (PD-L1 TPS ≥1%). 2

Table S2. Exposure-Adjusted AE Rates for Treatment-Related AEs That Occurred in ≥ 10% of Participants in Either Treatment Group. 3

Table S3. Exposure-Adjusted Treatment-Related AE Rates for Immune-Mediated AEs and Infusion Reactions. 6

Figure S1. OS Analysis in Key Subgroups in Participants With PD-L1 TPS ≥1%....................................... 8

Figure S2. Kaplan-Meier estimates of PFS in participants with (A) PD-L1 TPS ≥50%, (B) PD-L1 TPS ≥20%, (C) PD-L1 TPS ≥1%. 9

Figure S3. Kaplan-Meier estimates of PFS2 in participants with (A) PD-L1 TPS ≥50%, (B) PD-L1 TPS ≥20%, (C) PD-L1 TPS ≥1%. 11

Table S1. Subsequent Therapies in the ITT Population (PD-L1 TPS ≥1%).

Therapy	Pembrolizumab (n = 128)	Chemotherapy (n = 134)
Any subsequent oncologic therapy	80 (62.5)	79 (59.0)
Chemotherapy	67 (52.3)	47 (35.1)
Single agent (without bevacizumab)	19 (14.8)	16 (11.9)
Doublet (without bevacizumab)	8 (6.3)	13 (9.7)
Doublet (with bevacizumab)	4 (3.1)	8 (6.0)
Platinum doublet (without bevacizumab)	59 (46.1)	26 (19.4)
Platinum doublet (with bevacizumab)	6 (4.7)	1 (0.7)
Targeted therapy	17 (13.3)	20 (14.9)
Immunotherapy	10 (7.8)	35 (26.1)
Durvalumab	1 (0.8)	0
Nivolumab	1 (0.8)	14 (10.4)
Pembrolizumab[a]	3 (2.3)	11 (8.2)
Tagitanlimab	0	1 (0.7)
Other anti–PD-(L)1	4 (3.1)	11 (8.2)
Ipilimumab (± nivolumab)	0	2 (1.5)
Other immunotherapy	2 (1.6)	0
Other	7 (5.5)	7 (5.2)
Multi-Targeting Tyrosine Kinase Inhibitor	2 (1.6)	0

ITT, intent to treat; PD-(L)1, programmed cell death protein 1 or programmed cell death ligand 1; TPS, tumor proportion score.

[a]Excludes participants who received second-course pembrolizumab on-study.

Table S2. Exposure-Adjusted AE Rates for Treatment-Related AEs That Occurred in ≥ 10% of Participants in Either Treatment Group.

Adverse Event	Pembrolizumab (n = 128)	Chemotherapy (n = 125)
Increased alanine aminotransferase	22 (17.2)	27 (21.6)
Increased aspartate aminotransferase	22 (17.2)	23 (18.4)
Rash	17 (13.3)	5 (4.0)
Hypothyroidism	15 (11.7)	0
Pyrexia	15 (11.7)	10 (8.0)
Pruritus	14 (10.9)	2 (1.6)
Anemia	11 (8.6)	65 (52.0)
Fatigue	8 (6.3)	20 (16.0)
Decreased appetite	7 (5.5)	36 (28.8)
Decreased platelet count	4 (3.1)	28 (22.4)
Decreased neutrophil count	3 (2.3)	70 (56.0)
Leukopenia	3 (2.3)	16 (12.8)

Constipation	2 (1.6)	17 (13.6)
Nausea	2 (1.6)	23 (18.4)
Alopecia	1 (0.8)	32 (25.6)
Decreased white blood cell count	1 (0.8)	64 (51.2)
Vomiting	1 (0.8)	16 (12.8)
Neutropenia	0	14 (11.2)

**Observation period, mo**	**0–12**	**12–24**	**24–48**	**>48**	**0–12**	**12–24**	**24–48**	**>48**
Exposed at the start of interval, n	128	46	24	0	125	8	2	1
Total exposure[a], person-months	950.14	368.19	19.40	0	541.22	57.00	24.51	9.30
Total events (rate per 100 person-months)[b]	439 (46.20)	61 (16.57)	1 (5.15)	0	1373 (253.69)	62 (108.76)	7 (28.56)	5 (53.77)[c]
Increased alanine aminotransferase	32 (3.4)	5 (1.4)	0	0	47 (8.7)	2 (3.5)	0	0
Increased aspartate aminotransferase	28 (3.0)	2 (0.5)	0	0	37 (6.8)	2 (3.5)	0	0
Rash	20 (2.1)	0	0	0	6 (1.1)	0	0	0
Hypothyroidism	16 (1.7)	1 (0.3)	0	0	0	0	0	0
Pyrexia	24 (2.5)	0	0	0	10 (1.9)	0	0	0

Pruritus	21 (2.2)	5 (1.4)	0	0	2 (0.4)	0	0	0
Anemia	8 (0.8)	5 (1.4)	0	0	103 (19.0)	4 (7.0)	1 (4.1)	1 (10.8)
Fatigue	11 (1.2)	0	0	0	37 (6.8)	0	0	0
Decreased appetite	8 (0.8)	0	0	0	65 (12.0)	0	0	0
Decreased platelet count	5 (0.5)	3 (0.8)	0	0	79 (14.6)	9 (15.8)	0	0
Decreased neutrophil count	2 (0.2)	1 (0.3)	0	0	222 (41.0)	10 (17.5)	0	0
Leukopenia	2 (0.2)	2 (0.5)	0	0	44 (8.1)	2 (3.5)	0	0
Constipation	2 (0.2)	0	0	0	18 (3.3)	0	0	0
Nausea	2 (0.2)	0	0	0	34 (6.3)	1 (1.8)	0	0
Alopecia	1 (0.1)	0	0	0	33 (6.1)	0	0	0
Decreased white blood cell count	1 (0.1)	0	0	0	229 (42.3)	10 (17.5)	0	0
Vomiting	1 (0.1)	0	0	0	18 (3.3)	2 (3.5)	0	0
Neutropenia	0	0	0	0	24 (4.4)	2 (3.5)	0	0

AE, adverse event.

Values are presented as n (%) unless noted otherwise.

[a]Drug exposure is defined as the interval of min (last dose date + 30, Cutoff Date) – first dose date + 1.

[b]Data show AEs and include multiple occurrences of events.

[c]1 participant had 5 events.

Table S3. Exposure-Adjusted Treatment-Related AE Rates for Immune-Mediated AEs and Infusion Reactions.

Immune-Mediated AEs and Infusion Reactions,[a] n (%)	Pembrolizumab n = 128				Chemotherapy n = 125			
Any	34 (26.6)				7 (5.6)			
Hypothyroidism	15 (11.7)				0			
Pneumonitis	10 (7.8)				0			
Hyperthyroidism	7 (5.5)				0			
Infusion reactions	4 (3.1)				7 (5.6)			
Hepatitis	2 (1.6)				0			
Severe skin reactions	2 (1.6)				0			
Thyroiditis	2 (1.6)				0			
Colitis	1 (0.8)				0			
Hypoparathyroidism	1 (0.8)				0			
Pancreatitis	1 (0.8)				0			
**Observation period, mo**	**0–12**	**12–24**	**24–48**	**>48**	**0–12**	**12–24**	**24–48**	**>48**

Exposed at the start of interval, n	128	46	24	0	125	8	2	1
Total exposure[a], person-months	950.14	368.18	19.38	0	541.21	57.00	24.51	9.30
Total events of immune-mediated AEs (rate per 100 person-months)[b]	46 (4.84)	7 (1.90)	0	0	8 (1.48)	0	0	0
Hypothyroidism	16 (1.7)	2 (0.5)	0	0	0	0	0	0
Pneumonitis	12 (1.3)	1 (0.3)	0	0	0	0	0	0
Hyperthyroidism	7 (0.7)	2 (0.5)	0	0	0	0	0	0
Infusion Reactions	4 (0.4)	0	0	0	8 (1.5)	0	0	0
Hepatitis	1 (0.1)	1 (0.3)	0	0	0	0	0	0
Severe Skin Reactions	2 (0.2)	0	0	0	0	0	0	0
Thyroiditis	2 (0.2)	0	0	0	0	0	0	0
Colitis	0	1 (0.3)	0	0	0	0	0	0
Hypoparathyroidism	1 (0.1)	0	0	0	0	0	0	0
Pancreatitis	1 (0.1)	0	0	0	0	0	0	0

[a]Events were based on a list of terms specified at the time of analysis and were included regardless of attribution to study treatment or immune relatedness by the investigator. Related terms were included.

[b]Drug exposure is defined as the interval of min (last dose date + 30, Cutoff Date) – first dose date + 1.

[c]Data show AEs and include multiple occurrences of events.

Figure S1. OS Analysis in Key Subgroups in Participants With PD-L1 TPS ≥1%. OS, overall survival; PD-L1, programmed cell death ligand 1; TPS, tumor proportion score.

Subgroup	No of Events/ No of Participants	HR for OS (95% CI)
**Overall**	220/262	0.66 (0.51-0.87)
**Age**		
<65 years	147/172	0.60 (0.43-0.84)
≥65 years	73/90	0.79 (0.47-1.31)
**Sex**		
Female	29/38	0.78 (0.34-1.77)
Male	191/224	0.63 (0.47-0.85)
**ECOG PS**		
0	46/60	0.58 (0.32-1.07)
1	174/202	0.70 (0.52-0.95)
**Histology**		
Squamous	123/147	0.65 (0.45-0.93)
Nonsquamous	97/115	0.70 (0.46-1.05)
**Smoking status**		
Never	48/59	0.76 (0.42-1.38)
Former	124/146	0.58 (0.40-0.85)
Current	48/57	0.75 (0.41-1.36)
**Investigator's choice of chemotherapy**		
Pemetrexed and carboplatin	87/103	0.80 (0.52-1.23)
Paclitaxel and carboplatin	133/159	0.59 (0.41-0.84)
**Disease status**		
Metastatic	176/208	0.70 (0.51-0.95)
Locally advanced	44/54	0.45 (0.22-0.92)
**Baseline tumor size**		
At/above median	103/119	0.56 (0.36-0.89)
Below median	117/142	0.97 (0.66-1.44)

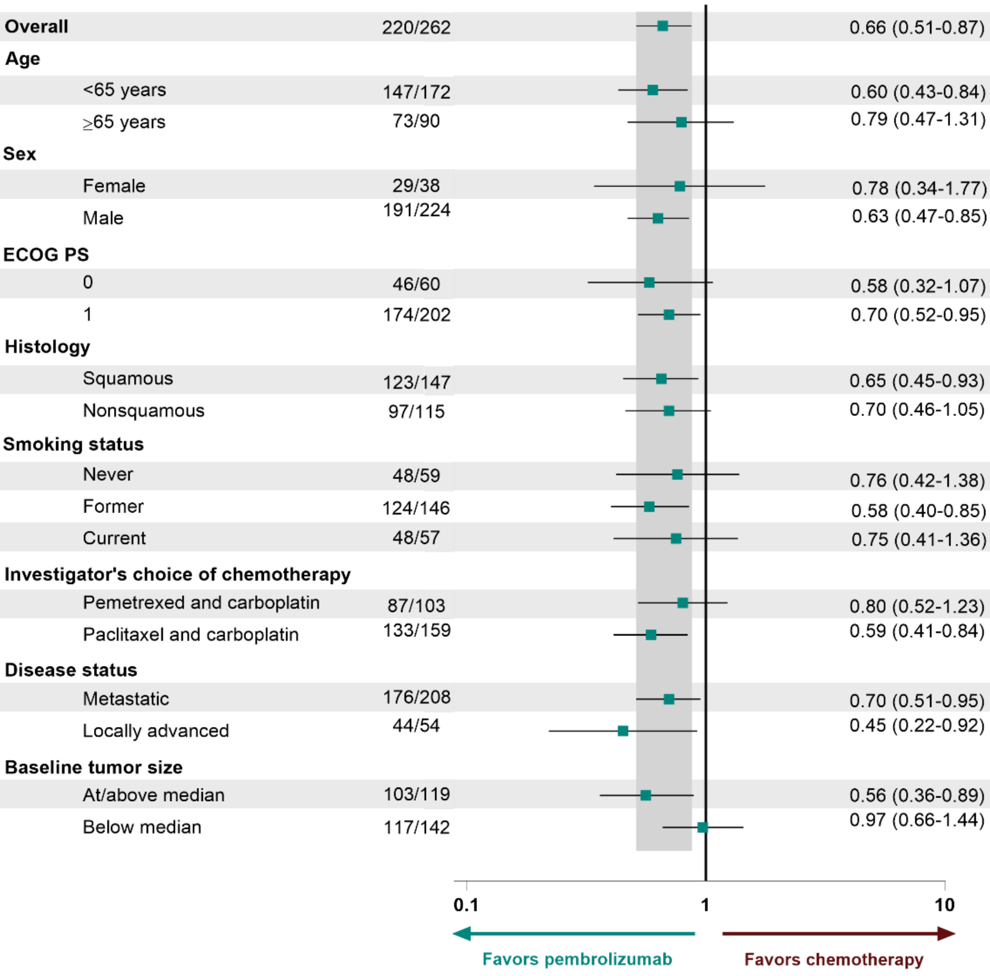

Figure S2. Kaplan-Meier estimates of PFS in participants with (A) PD-L1 TPS ≥50%, (B) PD-L1 TPS ≥20%, and (C) PD-L1 TPS ≥1%. PD-L1, programmed death ligand 1; PFS, progression-free survival; TPS, tumor proportion score.

(A) PFS of TPS ≥50%

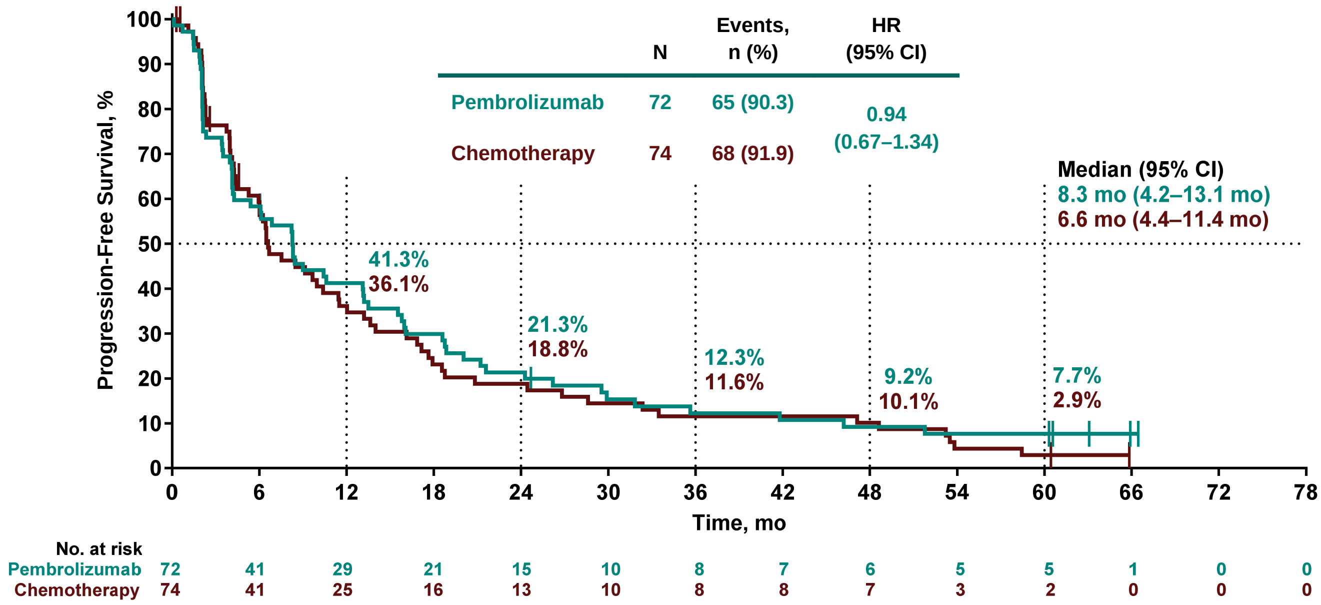

(B) PFS of TPS ≥20%

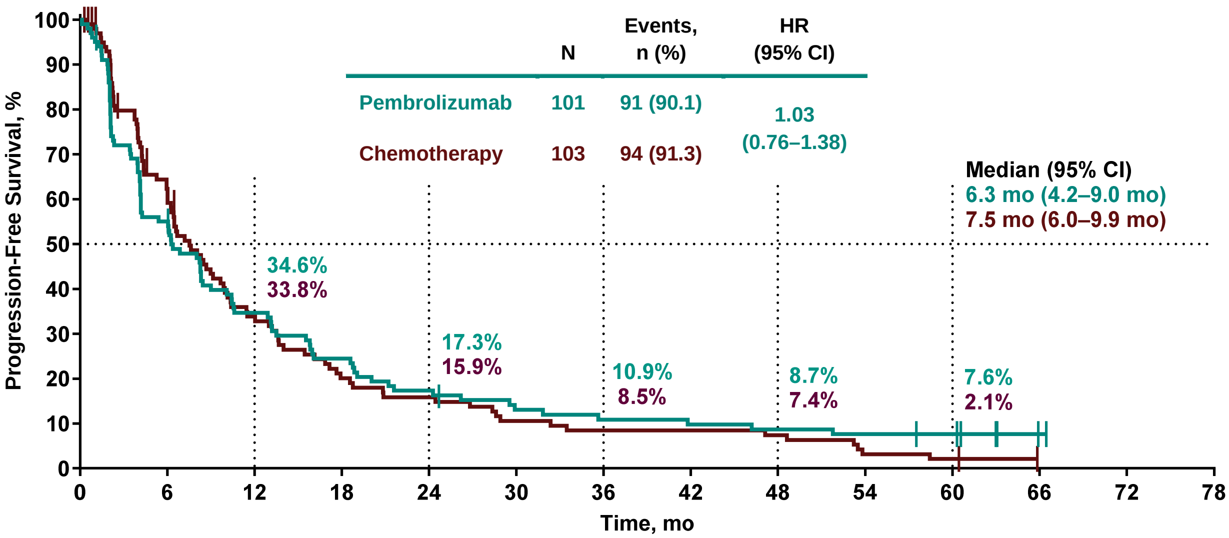

No. at risk														
Pembrolizumab	101	54	34	24	17	12	10	9	8	7	6	1	0	0
Chemotherapy	103	60	32	19	15	10	8	8	7	3	2	0	0	0

(C) PFS of TPS ≥1%

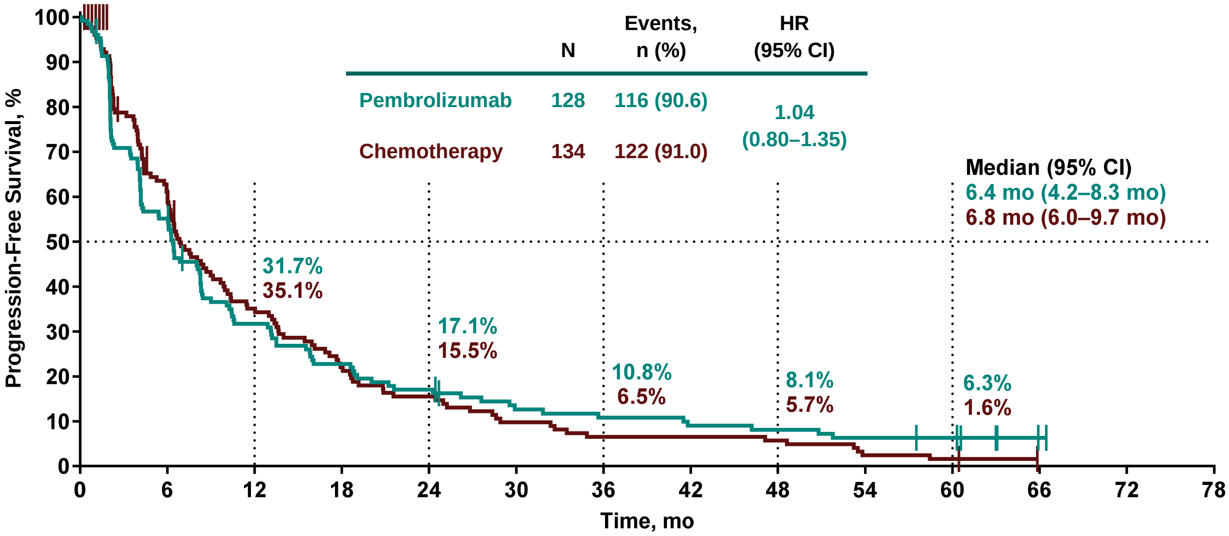

No. at risk														
Pembrolizumab	128	69	39	28	21	14	12	10	9	7	6	1	0	0
Chemotherapy	134	76	43	27	19	12	8	8	7	3	2	0	0	0

Figure S3. Kaplan-Meier estimates of PFS2 in participants with (A) PD-L1 TPS ≥50%, (B) PD-L1 TPS ≥20%, and (C) PD-L1 TPS ≥1%. PD-L1, programmed death ligand 1; PFS, progression-free survival; TPS, tumor proportion score.

(A) PFS2 in Participants with PFS of TPS ≥50%

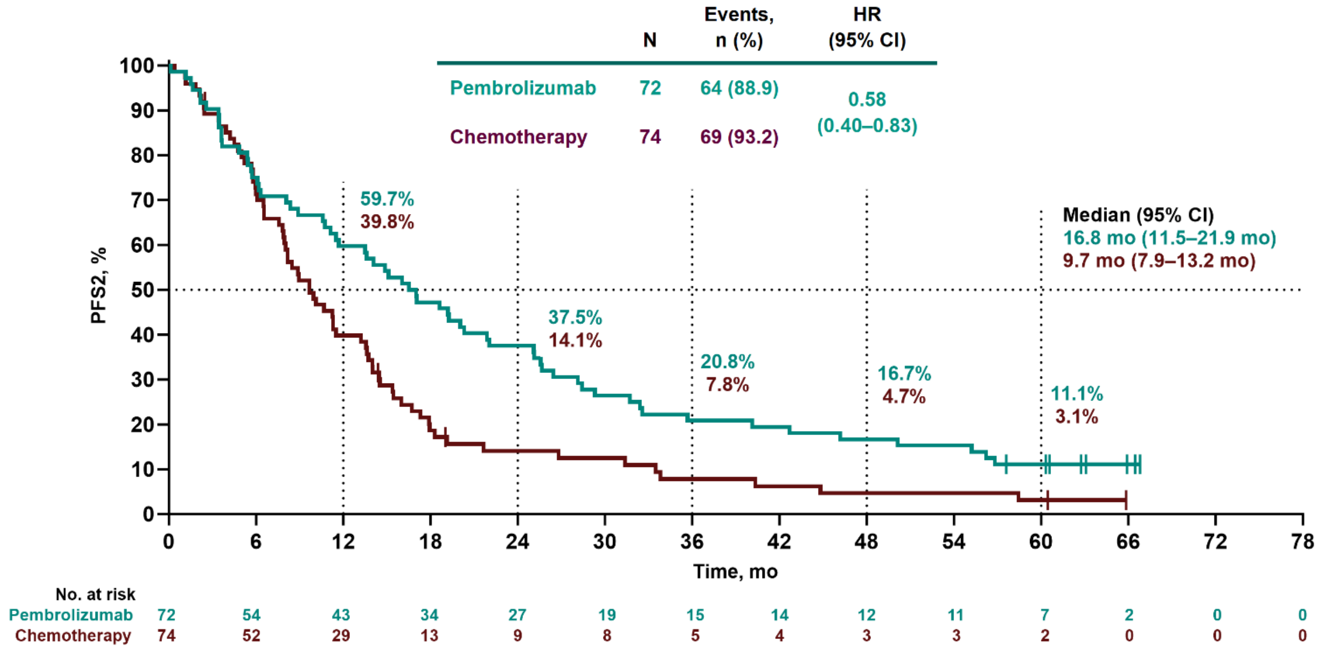

(B) PFS2 in Participants with PFS of TPS ≥20%

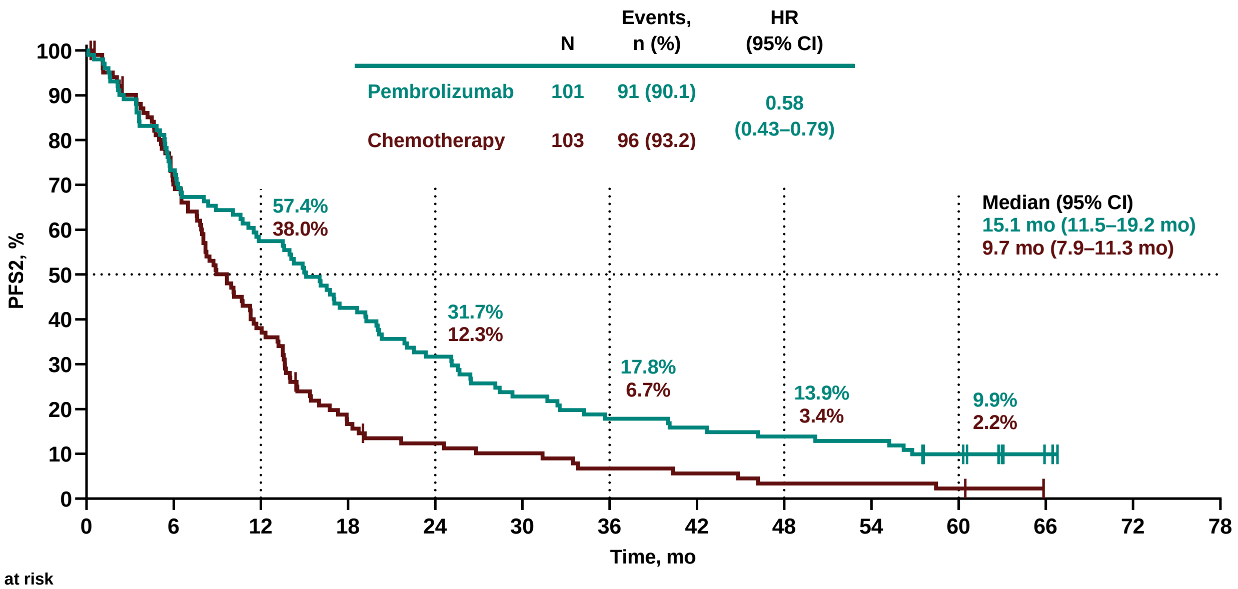

No. at risk														
Pembrolizumab	101	74	58	43	32	23	18	16	14	13	8	2	0	0
Chemotherapy	103	70	38	16	11	9	6	5	3	3	2	0	0	0

(C) PFS2 in Participants with PFS of TPS ≥1%

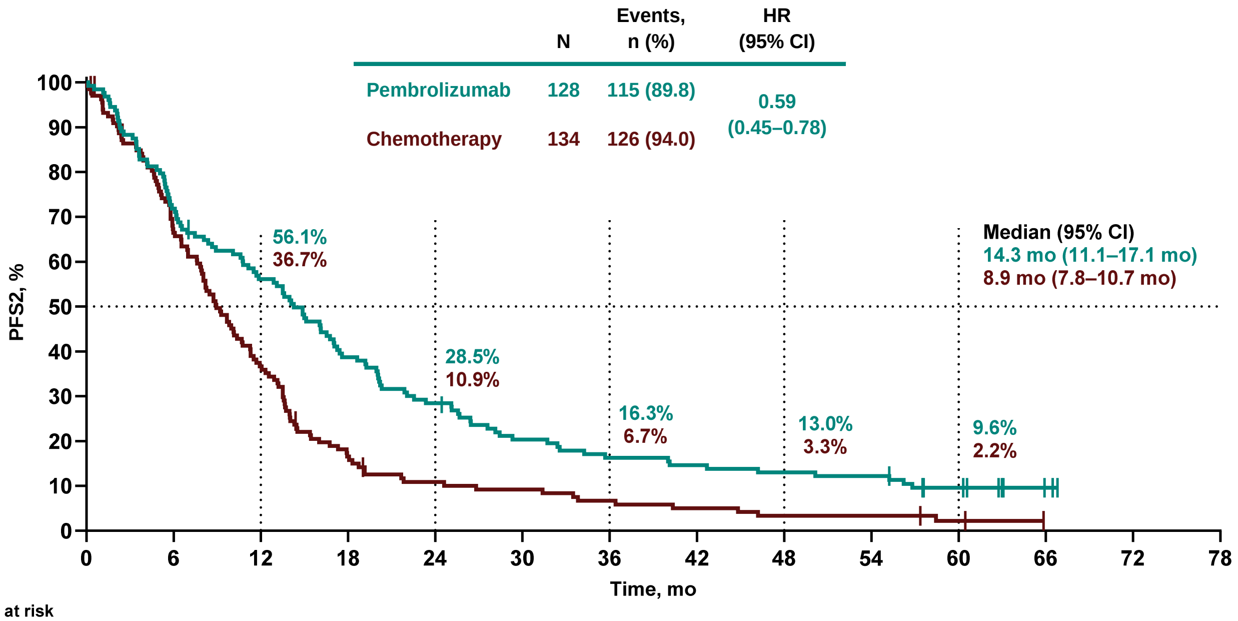

No. at risk														
Pembrolizumab	128	92	71	49	36	25	20	18	16	15	9	3	0	0
Chemotherapy	134	87	48	21	13	11	8	6	4	4	2	0	0	0
